# Nature's Electric Potential: A Systematic Review of the Role of Bioelectricity in Wound Healing and Regenerative Processes in Animals, Humans, and Plants

**DOI:** 10.3389/fphys.2017.00627

**Published:** 2017-09-04

**Authors:** Sheena E. B. Tyler

**Affiliations:** John Ray Research Field Station Cheshire, United Kingdom

**Keywords:** regeneration, wound current, voltage mapping, electric field, *V*_mem_

## Abstract

Natural endogenous voltage gradients not only predict and correlate with growth and development but also drive wound healing and regeneration processes. This review summarizes the existing literature for the nature, sources, and transmission of information-bearing bioelectric signals involved in controlling wound healing and regeneration in animals, humans, and plants. It emerges that some bioelectric characteristics occur ubiquitously in a range of animal and plant species. However, the limits of similarities are probed to give a realistic assessment of future areas to be explored. Major gaps remain in our knowledge of the mechanistic basis for these processes, on which regenerative therapies ultimately depend. In relation to this, it is concluded that the mapping of voltage patterns and the processes generating them is a promising future research focus, to probe three aspects: the role of wound/regeneration currents in relation to morphology; the role of endogenous flux changes in driving wound healing and regeneration; and the mapping of patterns in organisms of extreme longevity, in contrast with the aberrant voltage patterns underlying impaired healing, to inform interventions aimed at restoring them.

## Introduction

Regenerative medicine strategies aim to facilitate the healing processes, or provide replacements for diseased or damaged tissues resulting from birth defects, cancer, traumatic injury, degenerative diseases, and impaired wound healing (Levin, 2012a, 2013; Bessonov et al., 2015; Gurtner and Chapman, 2016). However, translation of regenerative strategies into effective clinical outcomes still remains a major challenge. For instance, chronic wound management costs £328.8 million per year in Wales, representing £1,727 per patient (Phillips et al., 2015). With treatment in specialized wound centers in Germany these costs rise to £9,569 per patient (Purwins et al., 2010). One in five in-patients in European hospitals have a pressure ulcer (Martin-Granados and McCaig, 2014)—which are open wounds that are exceptionally difficult to heal, often progressing to become life-threatening (Kuffler, 2015). Moreover, in spite of medical interventions there is a rising prevalence of such wounds, associated with the increasing incidence of diabetes mellitus, and an increasingly aging population (Fonder et al., 2008), with 15% of diabetic foot ulcers resulting in amputation (Posnett and Franks, 2008), or an amputation each 30 s worldwide (Richard and Schuldiner, 2008), which in turn leads to over 50% mortality within 5 years (Reiber et al., 1995). What is more, clinical regenerative techniques are hampered by a lack of knowledge of fundamentally how these processes are co-ordinated at both molecular and supra-cellular levels (Eming et al., 2014; Tosenberger et al., 2015). Thus, discovery of the key underlying mechanisms by which lost or damaged tissues are replaced remains an important research field.

Bioelectricity refers to the flow of electrical currents, carried by mobile charged ions, across cell membranes and along the exterior and interior ionic environment of cells (Mitcheson and Stanfield, 2013). Bioelectric phenomena are known to be associated with wound healing in animals (Nuccitelli, 2003, McCaig et al. (2009), Reid and Zhao (2014), and plants (Rhodes et al., 1996; Christmann and Grill (2013). Highly regenerating animals such the salamander can regenerate lower jaws, hearts, limbs, and brain tissue (Agata and Inoue, 2012). Compared with animals, plants in general have an even higher regenerative capacity (Ikeuchi et al., 2016), and plants such as *Acetabularia* can regenerate apical whorl form from even enucleated apical stalks (Mandoli, 1998). Moreover, a diverse range of bioelectric phenomena are central to these processes (Novak and Sironval, 1975; Borgens et al., 1977a, 1983).

The following is a review of published literature relating to the roles of bioelectric phenomena in wound healing and regeneration across the animal and plant kingdoms.

Firstly, an overview is provided to compare the bioelectric mechanisms of wound healing in both kingdoms. Common principles have been previously been distilled from such comparisons (Birnbaum and Alvarado, 2008). However, common regenerative strategies may result from deeply divergent mechanisms. Knowledge is thus needed to prevent researchers pursuing common ground that does not exist between the kingdoms, or if the divergences are so fundamental as to preclude true translational relevance. Therefore, it is useful to probe the realistic limits of mechanistic similarities between the kingdoms. Secondly it is demonstrated how insights from the bioelectric mechanisms in animal and plants have led to clinical applications for wound healing. Finally, it is suggested how further such insights from animals and plants can indeed provide promise to inform future research strategies.

## Materials and method

A systematic search of the literature from 1980 to 2017 was performed using information-retrieval tools including the search engines PubMed, PubMed Central and Biosis (Web of Science), Science Direct and Google Scholar. Combinations of keywords comprised the terms bioelectric, membrane voltage, endogenous electric field, applied electric field, bioelectromagnetic, V-ATPase, wound current, wound healing, regeneration, animal, plant, and human. In addition, papers of historical importance not indexed in the above databases were obtained by study of key publications cited in this review, such as Levin (2003), Nuccitelli (1988), and Rosene and Lund (1953). Following Levin (2003) such papers were selected on the basis of quality, importance of result, or novel discoveries. Clinical studies were primarily assessed according to criteria in the Cochrane Handbook for Systematic Reviews of Interventions (www.cochrane.org). This was to maximize inclusion of the highest levels of evidence (as, for instance, in Koel and Houghton, 2014), but also permitted lower levels of evidence, such as from case studies, where the data appeared robust and well-authenticated. Excluded from this review were neuronal action potentials, electromagnetic field detection using SQUID and ECG, and potential health effects from extremely low frequency electric and magnetic fields, all for which there is already a large literature and is not within the scope of this review topic.

## Results and discussion

### Bioelectric mechanisms of wound healing and regenerative processes in animals and plants—an overview

**Figure 1:**
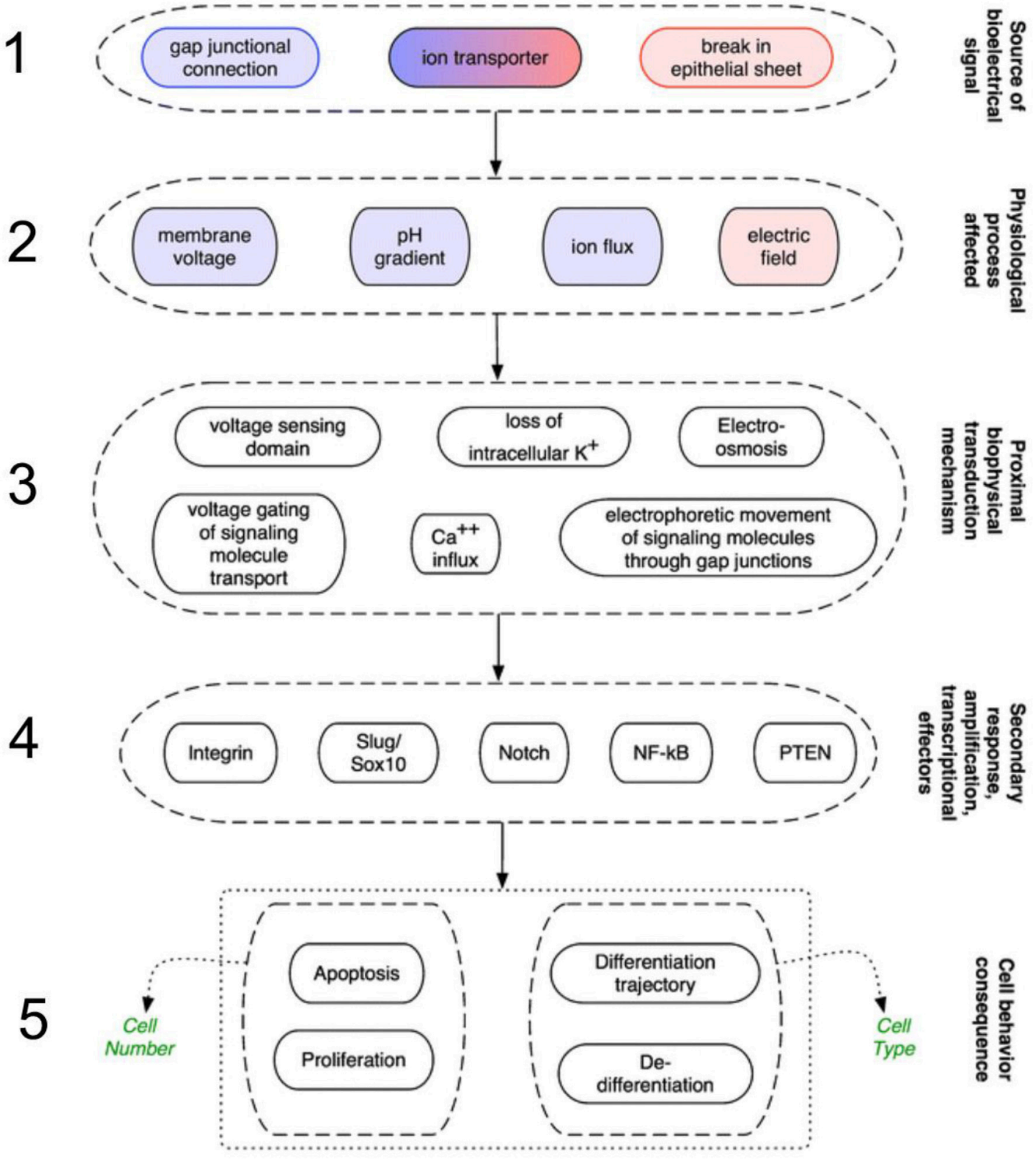
Mechanisms in animals that transduce electrical signals into second-messenger cascades through a series of sequential phases. 1. Signals arrive via gap junction connections, or are initiated from variations in ion channels/pumps, or leaks across wounded cells/cell layers. 2. These signals are carried by membrane potential changes, pH gradients, fluxes (flow of ions), or electric fields. 3. Next, various mechanisms act as biophysical receptors for these signals. 4. The signals activate a number of genetic signaling pathways such as integrin and Slug/Sox10. 5. The resulting transcriptional cascades orchestrate changes in cell behavior (such as proliferation and differentiation), and remote tissues, enabling wounds to discern what already exists and what must be recreated (from Sundelacruz et al., 2009, reprinted with permission).

Throughout the plant and animal kingdoms, voltage gradients predict, and correlate with growth and patterning events, forming co-ordinates to provide morphogenetic cues (reviewed by, for instance, Burr, 1947; Levin, 2003; McCaig et al., 2005). Information-bearing signals are integrated with a cascade of cellular responses in a number of sequential phases of a scheme proposed for animals (Levin, 2009; Sundelacruz et al., 2009; Figure 1). In the first phase, the initial source of electrical signals emanate from spatial variations in ion channels or pumps such as V-ATPases, leaks across wounded cells or cell layers, or may arrive via gap junction connections (McCaig et al., 2005; Levin, 2007). The resulting ionic gradients drive extracellular ionic current flow, and this establishes the voltage gradients (McCaig et al., 2009). In the second phase, although they are physically inter-related, the signals can be carried by any of a number of entities: an electric field (which is the presence of dipole with no immediate barrier); membrane potential (the presence of dipole across a barrier with selective permeability created by ion gradients across membrane via actions of ion channels and pumps); fluxes (flow of ions through channels or pumps per unit time); and pH gradients (represented by proton pumping to modify H^+^ gradients). The third phase comprises the mechanisms acting as receptors for these signals. Fourthly, these in turn activate downstream a number of gene responses, which in the fifth phase evoke transcriptional cascades involved in the control of morphogenesis and regeneration (Sundelacruz et al., 2009). Plants would diverge from this scheme in a number of respects. For instance, the intercellular connections are represented by the symplastic network, by which cells become electrically coupled (Rinne and van der Schoot (1998); and wound-generated electrical signals (e.g., in the form of membrane depolarizations) activate jasmonate-mediated gene expression and plant-specific downstream cascades (Christmann and Grill, 2013).

How might these bioelectric signals bear body patterning information? Levin (2012b) indicates that it has been recognized for over 60 years how “spatial patterns of bioelectric parameters (e.g., voltage differences between specific locations) quantitatively predict” anatomical outcomes. These observations led Burr, Sinnott and others to propose that the biological patterns of life are the “visible expression of an underlying bioelectrical pattern” (Sinnott, 1960). Levin adds (2013) that “bioelectric patterning information can be dynamically written and read from,” envisaged as a prepattern (i.e., templates of shape), to generate anatomical structures (Levin, 2012a). A source for this pattern has been suggested from evidence of “bioelectric patterning during oogenesis” in *Drosophila*, in which follicle cell *V*_*mem*_ and pH patterns can be attributed to an asymmetric distribution of V-ATPases (proton pumps; Krüger and Bohrmann, 2015).

D'Arcy Thompson showed how body form can be described in terms of a set of geometrical co-ordinates. By subjecting the net to simple mathematical, or so-called Cartesian transformations, related living species could be generated (Thompson, 1942). Thompson recognized that a comprehensive “law of growth” pervaded the whole body structure, in which a recognizable system of forces was at work (Thompson, 1942). It can be envisaged that these geometrical co-ordinates are provided by three-dimensional gradients of voltage (Shi and Borgens, 1995). Other authors have suggested that the body pattern of organization is established by complex pattern of electric fields (Burr and Northrop, 1937, 1939; Levin, 2003). In both variants (3D voltage network or field system), it is the dynamic state of bioelectric circuits that determine the functional signaling properties of physiological networks, which in turn have patterning outcomes (Levin, 2014; Sullivan et al., 2016). The following examples provide evidence of patterning outcomes attributable variously to membrane voltages, current flows, or electric field patterns. In plants, externally measured voltage differences in Douglas fir conform to the complex tree morphology (Rosene and Lund, 1953); electric currents precede lateral root emergence (Hamada et al., 1992); and the electric field (hereafter EF) patterns in fruits correlate with the development of their morphology (Burr and Sinnott, 1944); meristematic tissue of turnip seedling shoots and maize seedling roots is electrically negative to surrounding differentiated tissue (McAulay et al., 1951); in eggs of the brown alga *Pelvetia*, the site of inward current predicts the germination site, from which the body axis of polarity is established (Nuccitelli, 1978); and daily and annual rhythms of extracellular electric potentials are related to tree cambial growth (Fensom, 1963; Fromm and Lautner, 2007). In animals, endogenous electric fields, generated by an apical-basal current in polarized epithelial cells, are implicated during cleavage of mouse embryos (Nuccitelli and Wiley, 1985). The ion current pattern in mollusc embryos correlates with the embryo polarity and oscillates with the cell cycle during cleavage (Zivkovic et al., 1991a), reverses direction prior to during mesoderm induction (Zivkovic et al., 1991b), and may drive migration of cell populations during organogenesis (Créton et al., 2002). Voltage gradient patterns guide chick tail development (Hotary and Robinson, 1992), correlate with embryo growth in salamanders (Burr and Hovland, 1937), and predict the form of the amphibian neurula (Borgens et al., 1983), cranio-facial morphology (Vandenberg et al., 2011) and limb bud location prior to any anatomical development there (Borgens, 1983; Borgens et al., 1983). Bioelectric coupling via intracellular channels (gap junctions) and local field potentials between cells (Pietak and Levin, 2016) underlie changes in physiological networks driving species-specific differences in the form of planarian worms (Emmons-Bell et al., 2015). It can be difficult to distinguish which aspects of ion flow bear the instructive signals (i.e., whether *V*_*mem*_ change, electric field, or ion flow), particularly since there is a co-ordinated interaction between these entities (Krüger and Bohrmann, 2015), but in some cases this has been achieved, as in misexpression of electroneutral transporters, which distinguish between the importance of voltage changes and specific ion flux (Sundelacruz et al., 2009).

Ubiquitously throughout these kingdoms, this bioelectric signaling system provides instructive information ranging from the sub-cellular, with microdomains bearing distinct voltage characteristics (Martens et al., 2004), to field patterns evident at the whole embryo level (Jaffe, 1981; Nuccitelli, 1984), and do not merely reflect developmental processes but have a determinative role. For instance, this is evidenced by modifying the endogenous field, which leads to changes in the body axis orientation of fucoid eggs (Robinson and Cone, 1980), and induces ectopic eye formation in *Xenopus* embryos (Pai et al., 2012). An applied EF results in a reversal of the normal polarity of morphology in *Obelia* (Lund, 1921), and in planarian worms leads to head structures at the tail end and *vice versa* (Marsh and Beams, 1957). On wounding, there is a dramatic reawakening of the tissue building machinery (Martin and Parkhurst, 2004), with some of the same genes and signaling molecules functioning in wound healing and regeneration as they do in early embryonic development (Bryant et al., 1987). For instance, amputated digits of mice express the developmental gene *Bmp4*, which is upregulated during tip regeneration (Han et al., 2008). In both animals and plants, bioelectric signatures active during development also have a central place in the initial wound response, and appear to drive the wound healing process (Davies, 1987, 2004; Nuccitelli, 2003). Characteristics shared in both kingdoms include the generation of electric current immediately on wounding at the wound site (Geddes and Hoff, 1971; Stankovic et al., 1998; McCaig et al., 2005; Zimmermann et al., 2009); and the involvement of ion channels in this process.

Both kingdoms also employ numerous regeneration strategies in common (Birnbaum and Alvarado, 2008). One strategy involves turning back the clock on differentiation, to overcome the barriers to totipotency. Sometimes this results in production of a massive growth of cells associated with wounding—the blastema in animals (Han et al., 2005; McCusker et al., 2015), and the callus in plants (Ikeuchi et al., 2013). According to Birnbaum and Alvarado (2008), callus is a tissue mass in which mature, differentiated cells become dedifferentiated. However, callus histology resembles the primordia of lateral roots (Atta et al., 2009), with gene expression profiles similar to that of root meristems, indicating that callus tissue is not fully dedifferentiated (Sugimoto et al., 2010). Another strategy is the maintenance and recruitment of adult stem cells. In plants the adult plant meristems, comprising stem cell niches, continually generate the various cell types and major axes (Dinneny and Benfey, 2008). The net result of these regeneration strategies is that, in both kingdoms, the blastemal and callus cells exhibit a stem cell-like state with the ability to grow, divide and subsequently differentiate into the required new tissue types. A role for bioelectric mechanisms in these processes is implied by electrical manipulation of the callus (Section Mapping of Voltage Patterns in Body Patterning and Wound Healing), which is thought to re-align the physiological polarities of the callus cells (Goldsworthy, 1996; Carmen, 2006), and perturbation of the endogenous electric current associated with the regeneration blastema (Monteiro et al., 2014; Section The Role of Ionic Flux Changes in Highly Regenerating Animals).

Moreover, both plant and animal genomic DNA is organized into euchromatin and heterochromatin, comprising highly conserved histone proteins and posttranslational modification machinery (Costa and Shaw, 2007). Ubiquitously in both kingdoms the cytoskeleton and DNA are electrically conductive (Tuszynski et al., 2004; Priel et al., 2006; Merino et al., 2008; O'Brien et al., 2017), such that it is conceivable that electrical signaling from the extracellular matrix, transduced by voltage-gated mechanisms, can be conducted continuously along cytoskeletal elements to DNA (McCaig et al., 2009), leading to chromatin remodeling, which is implicated in redirection of gene expression and the recovery of totipotency in regeneration (Birnbaum and Alvarado, 2008). Although there is a gap between bioelectric effects and their molecular biophysics basis, this is beginning to be bridged by Hekstra et al. (2016). Their protein model system demonstrates electric field-induced conformational changes, which are known to be central to many biological processes, including those of developmental significance (such as DNA replication).

In plants, an initial wound signal at the wound site leads to the generation of electrical signals (Davies, 1987): firstly of a slow wave of negativity known as a variation potential (VP); secondly membrane depolarizations self-propagated according to all-or-none properties, known as action potentials (APs); and thirdly hyperpolarizing system potentials (SPs; Zimmermann et al., 2009), which vary in intensity according to the stimulus (Mousavi et al., 2013). These lead to plant-specific effects including local hydraulic pressure changes and a systemic electric potential traveling primarily in the phloem, which in turn activates defense signal transduction cascades. In contrast, the bioelectric feature in animals is essentially a local, sustained wound current, which leads to the galvanotactic migration of epithelial cells and other cell types (such as neutrophils, lymphocytes, monocytes, macrophages, vascular endothelial cells, neurons, and fibroblasts) from the wound edge into the wound bed (reviewed by Martin-Granados and McCaig, 2014; Reid and Zhao, 2014). Plant cells in general are unable to migrate, and thus the plant wound callus develops and grows through successive cell divisions, which in trees may take several years to complete (Zajączkowska, 2014). Moreover, the molecular pathways [e.g., cdc42p and Rho/Rac in animals (Zhao et al., 2006), and the jasmonate pathway in plants (Mousavi et al., 2013)] and substrates employed in generating the wound plugs (Furch et al., 2007, 2009) are vastly different between the kingdoms, although membrane voltages are evident in both processes. Thus, the end result—the plug—is achieved in both kingdoms, but by completely different means.

However, one of the most fascinating similarities between wounding in animal and plants is the phenomenon of ionic flux changes. In plants an outward-to-inward current switch is associated with wound sites known in roots (Miller et al., 1988); in animals a transepithelial potential (TEP) collapses at the wound site and the TEP in the surrounding intact epithelium generates a flux directed into the wound region (Nuccitelli, 2003; McCaig et al., 2009). In highly regenerating animals, the regeneration current following amputation is characterized by a current reversal, in contrast to that of non-regenerating animals (Reid et al., 2009)—a point which is discussed later.

Plants, humans and animals also appear to share an electric signaling system comprising a neuromotoric apparatus and neurotransmitter-like substances. Specific systems described are glutamate-receptor-like (GLR) ion channels (Mousavi et al., 2013); cellular messengers such as calmodulin, and cellular motors (Baluška et al., 2006; Murch, 2006); voltage-gated ion channels (Galle et al., 2014); and synaptic activity (Baluška et al., 2006; Baluška and Mancuso, 2013). Therefore, study of common characteristics and behavior may illuminate molecular details of their modes of action. This is of clinical relevance. Neuronal injury and loss associated with Alzheimer's disease correlate with a legion of pathological changes in the glutamate system, such as degeneration of glutamatergic neurons and aberrant activation of glutamate receptors (Revett et al., 2013). Plant GLRs are intrinsic to the wound response: *Arabidopsis* plants defective in ion pumps and channels, resulting from loss of function of GLR genes, had impaired wound-induced signaling (Christmann and Grill, 2013). Mammalian neurotransmitter homologs are thought to share a modular structure in common with plants, comprising an amino-terminal domain, a ligand-binding domain, a transmembrane domain, and a carboxyl-terminal domain (Davenport, 2002). However, the mammalian neuromotoric system is deeply complex, and anatomically and physiologically fundamentally distinct from plants. For instance, the mammalian synapse exhibits thousands of distinct proteins, resulting in synapses possessing a high degree of molecular diversity (Granger et al., 2011; O'Rourke et al., 2012). Changes in transcription, translation and post-translational modifications provide further diversity to these proteins. Postsynaptic response transduction involves complex molecular cascades beyond the receptors themselves, with chaperones, kinases, and receptor modulators that also impart yet further functional diversity (Béïque et al., 2006; Kato et al., 2010; O'Rourke et al., 2012; Huganir and Nicoll, 2013). Together these characteristics contribute to the function of neural circuits, and show a limited homology with the molecular cascades and synaptic features of plant systems.

### The role of ionic flux changes in highly regenerating animals

Animal models have contributed to understanding the significance of voltage changes attending wound healing and regeneration, leading to the suggestion that the differences between regenerating and non-regenerating systems depends upon their bioelectric characteristics (Borgens et al., 1979b; Levin, 2003). Immediately upon wounding human skin, an endogenous ionic flux generates the so-called injury current, typically between 1 and 10 μA/cm^2^ (Nuccitelli, 1988, Figure 2). Amputation of digits or limbs leads to an even greater wound current flow of between 10 and 100 μA/cm^2^ in the region of remaining epidermis in amputated newt limbs (Borgens et al., 1977a). A current density of 22 μA/cm^2^ has been measured from the stumps of newly amputated human finger tips, which are able to regenerate, more usually in children (Illingworth and Barker, 1980).

**Figure 2:**
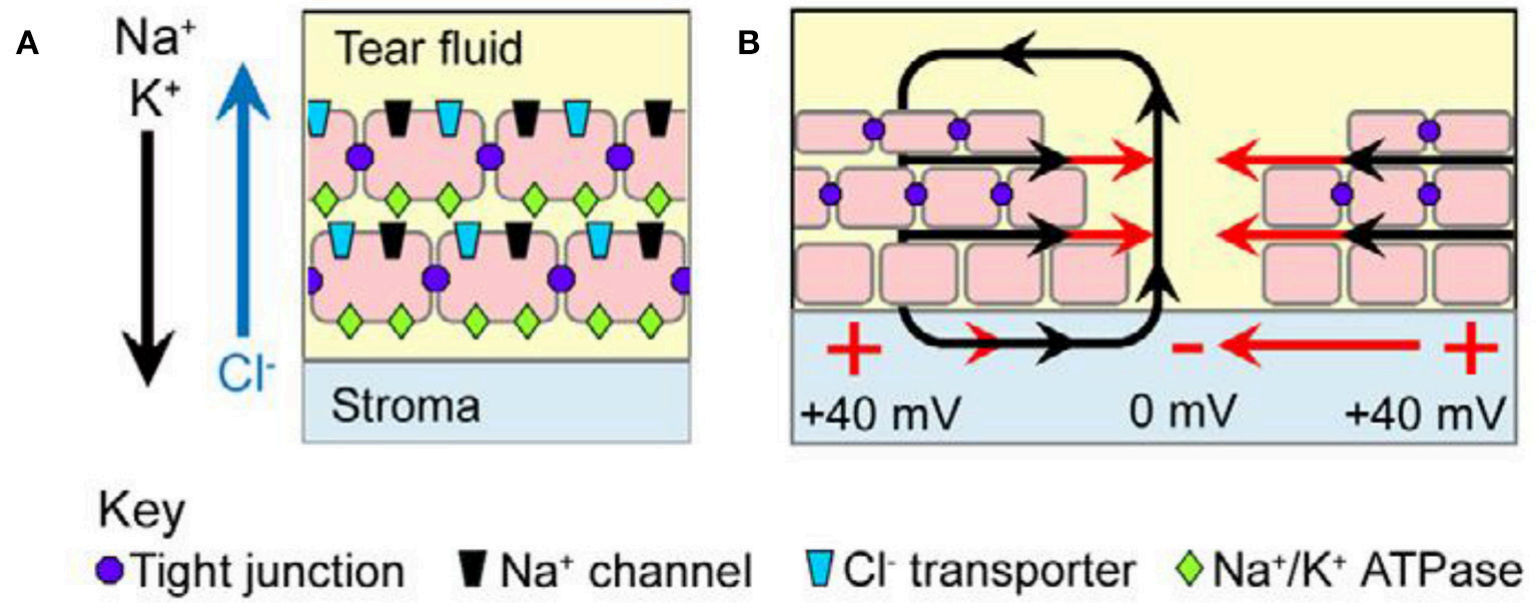
Wound current in the mammalian corneal epithelium model. **(A)** Tight junctions (purple dots) seal neighboring cells to each other and restrict lateral mobility of membrane proteins. The apical domain of each polarized cell is therefore enriched in Na^+^ channels (black) and Cl^−^ transporters (blue), whereas the basolateral domain contains Na^+^-K^+^ ATPases (green); this polarized distribution results in net movement of Na^+^ and K^+^ inward into the stromal layer and net movement of Cl^−^ outward into the tear fluid (arrows). The separation of charge across the tightly sealed epithelium results in a transepithelial potential (TEP) difference of ~40 mV, with the stroma positive relative to the tear fluid. **(B)** Upon injury, the TEP collapses to zero at the wound center, but it remains ~40 mV distally. This voltage gradient establishes an EF (red arrows) in the tissues parallel to the epithelial surface (from McCaig et al., 2009, reprinted with permission).

An active role for EFs in wound healing animal models is suggested by evidence from their enhancement or nullification (Messerli and Graham, 2011). Thus, disruption of endogenous EFs with pharmacological agents or applied EFs designed to reduce the wound current concomitantly retards wound healing (Rajnicek et al., 1988; Rhodes et al., 1990; Chiang et al., 1991), whilst interventions which strengthen the EFs accelerate the wound healing rate in injured bovine and rat cornea (Sta Iglesia and Vanable, 1998; Song et al., 2002).

Similarly in animal regeneration models, application of fields within the physiological range leads to regeneration of limb form and muscle, nerve and cartilage in amputated forelimbs of animals which do not normally regenerate them, such as *Xenopus* and *Rana* (Smith, 1967; Borgens et al., 1977b, 1979a), chicks (Sisken and Fowler, 1981) and rats (Becker, 1972). Repeating the Becker experiments more recently, in ES treated rat limb stumps the bone marrow cavity remained open, stimulating regeneration of osteocartilaginous and vascular tissue, possibly by causing bone marrow stem/progenitor cells to produce vascularized osseocartilaginous centers (Leppik et al., 2015). Amputated stumps of *Xenopus* tadpole tails can regenerate throughout development except during a “refractory period” at stage 45. Amputation induces large outward currents leaving the stump, but at stage 40 there is a large current reversal. Manipulation of this current alters regenerative ability (Reid et al., 2009). These various experiments suggest that endogenous stump currents play some causal role in initiating regeneration. Further evidence for a role of endogenous fields is found in cornea regeneration, in which the extent of neuronal regrowth correlates with the strength of an applied field (Song et al., 2004).

Moreover in animals with a high capacity for regeneration, such as salamanders, an initial event following limb amputation is the migration of cells over the wound surface to form the wound epithelium (Reid et al., 2009), which is essential for regeneration. By contrast, non-regenerating animals secrete an ECM to seal off the wound. Interestingly, this migratory response depends on the opposing activity of the PIP3/PTEN enzymes (reviewed by Stewart et al., 2007). In non-regenerating animals, the current following amputation has a positive polarity, and decreases slowly as the limb heals. In highly regenerating animals, this positive polarity later switches to a negative polarity, and the peak voltage correlates with the period of maximum cell proliferation (Borgens et al., 1984; Levin, 2009). Current leaves the stump end and returns in the skin of the limb axis. As in partially regenerating animals, disruption of these currents in amputated salamander and newt limbs with either ion blockers, removal of Na^+^ from the animals' pond water or reversing the EF leads to delayed or abnormal regeneration, confirming that the current is indeed required for regeneration (Borgens et al., 1979c; Jenkins et al., 1996; Levin, 2009). Reactive oxygen species (ROS), especially hydrogen peroxide, in conjunction with bioelectric activities are known to control the expression and activity of signaling pathways, such as Wnt, FGF, BMP, and Notch, during wounding and regeneration. It is interesting, therefore, that the current switch appears to be mediated by hydrogen peroxide upstream of voltage-gated Na^+^ channels (Ferreira et al., 2016). This brings a promising insight into how the biochemical and biophysical activities interact.

Proton pumping from the wound has been invoked as an instructive factor in these regeneration events. Regeneration in non-regenerating *Xenopus* tadpole tails can be triggered by artificially driving H^+^ efflux (Masuda and Montero-Lomeli, 2000), suggesting that the bioelectric signal is activating a positional information pathway or carries instructional information to guide the growth (Levin, 2012b; Tseng and Levin, 2013). Similarly, in the caudal fin of zebra fish, V-ATPases, which drive H^+^ efflux, are involved in the formation of position-dependent cues during regeneration (Monteiro et al., 2014). The high regenerative capacity of certain animals may be based on phylogenetically divergent mechanisms which may not directly translate to the mammal (Roshan and Grant, 2012). However, the above findings indicate that the failure of regeneration following organ loss or injury in humans, compared with highly regenerating animals, may be attributed at least in part to differences in bioelectricity—mediated signaling (Stewart et al., 2007). If so, bioelectric cues may be a key to improve clinical regenerative strategies, and the following evidence suggests that this is indeed the case.

### Clinical applications

#### Dermal wounds

Knowledge of the importance of bioelectric phenomena in the above wound healing models has provided the rationale that clinical application of electrical stimulation (ES) to chronic wounds would enhance healing. Typically ES is applied with either one electrode in the wound and the other one opposite to it, or with both electrodes around the wound (Koel and Houghton, 2014). This has met with a very promising success that has been recognized for decades, which emphasizes the huge relevance of the above studies to clinical wound healing and regenerative interventions. Houghton (2014) reviewed numerous well-designed clinical studies to evaluate the effect of various forms of ES therapy on human subjects with open wounds, resulting from aetiologies ranging from pressure ulcers and diabetic foot ulcers to leprosy. She concluded that monophasic pulsed current consistently demonstrated accelerated healing. Using very strict selection criteria, Koel and Houghton (2014) reviewed 15 high-quality studies of low frequency (<1,000Hz) ES, with either unidirectional and bidirectional (biphasic pulses) and also alternating EF that had employed the percentage area reduction (PAR) as a measure to compare wound healing rate. They concluded that ES is an evidence-based application with proven effectiveness, increasing wound area reduction by 40% in 4 weeks of treatment, i.e., ES almost doubles the healing rate. The results of unidirectional ES were better than for bidirectional ES.

ES is now approved for treatment of non-healing wounds by regulatory agencies in the EU such as the European Pressure Ulcer Advisory Panel (www.epuap.org), and the National Pressure Ulcer Advisory Panel, Washington DC (www.npuap.org/resources.html) in the United States (Houghton, 2014; Martin-Granados and McCaig, 2014). For instance, WoundEL®-therapy (Molnlycke) has been used with some success to treat more than 6,000 patients in Germany (Martin-Granados and McCaig, 2014). A recent device, a redox-active Ag/Zn bioelectric dressing, increases keratinocyte migration to facilitate wound closure, needs no external power source, conforms to skin topography and can be cut to the size of the wound (Banerjee et al., 2014). Similar wireless bioelectric dressings generate a micro-electrical field which may augment the natural electric field of injury following wounding (Kim et al., 2014), and have bactericidal activity on numerous wound pathogens including multidrug resistant organisms (Kim et al., 2016). In another study in chronic wounds unresponsive to conventional therapies, a bioelectrical signal therapy device generating an AC pulse train with a stochastic (random) signal led to 87% of the wounds undergoing closure (Fraccalvieri et al., 2015). In an appraisal for the National Health Service (NHS) in the UK, an externally applied electroceutical device was evaluated to be a cost-effective treatment for managing venous leg ulcers, with an improved outcome for less cost, compared with other treatments (Guest et al., 2015). In spite of such clinical promise, bioelectric interventions are often still not featured in wound healing conferences, and remain absent from many clinicians' repertoires, for reasons including lack of appropriate equipment and specialist/therapist education and training in ES application (Koel and Houghton, 2014). Nevertheless, Harding and colleagues maintain that ES technologies have a sound research underpinning (Harding, 2013), with the potential to become the mainstream intervention in the treatment of chronic and complex wounds (Clark, 2013).

The use of pulsed electromagnetic field (PEMF) therapy as another biophysical intervention is attractive because of its non-invasive characteristics (Chao and Inoue, 2003). In a randomized, double-blind, placebo-controlled clinical trial on chronic diabetic foot ulcers in 13 patients, PEMF (duration: 60 min; frequency: 12 Hz; intensity: 12 Gauss) accelerated wound healing and improved microcirculation (Kwan et al., 2015). Two case reports featured the use of Emysimmetric Bilateral Stimulation (EBS), a variant of PEMF, for untreatable skin ulcers (Guerriero et al., 2015). One of the cases concerned a 91 year old female presenting with painful, extensive, necrotic lower leg skin ulcers, which had been unresponsive to standard wound care. After 5 weeks of EBS treatment, the leg ulcers were completely healed. The authors also recognized the need for controlled further studies. EBS requires lower power stimulations compared with conventional PEMF, leading to potential and phase based interactions between the human body and extremely weak electromagnetic signals (Guerriero et al., 2015).

#### Fractures

Impaired healing of bone is another chronic wound-healing problem. PEMF, involving electromagnetic field changes, is a non-invasive application for fracture treatments, which may be less accessible to electric current applications. In a double-blind, randomized controlled trial study of PEMF treatment of 51 tibial shaft fractures, Sharrard (1990) concluded that radiologically, 50% of the active group healed compared to 8% of the control group. In a case study of a 75 year old Caucasian lady with recurrent and recalcitrant lower limb fractures, these remained mal-aligned and led to increasing pain following Manipulation under Anesthesia (MUA). She then received PEMF for at least 3 h per day until the fracture healed. Seven months later this resulted in complete bony union, with the patient fully weight-bearing and asymptomatic (Doorgakant et al., 2009). In a randomized, controlled study of 58 patients, PEMF treatment led to reduced suffering time between fracture and repair (Shi et al., 2013). In a systematic review of randomized controlled trials, PEMF significantly shortened the time to union for acute fractures of the upper limb (Hannemann et al., 2014). According to Sun et al. (2009) PEMF exposure increases the rate of bone fracture healing by causing human bone marrow mesenchymal stem cells (BMMSCs) to proliferate, providing an increased source of osteoblasts. PEMF also modifies the expression of genes implicated in osteogenesis (Sun et al., 2010). Upregulation of osteogenic genes was also proposed to explain positive clinical effects of capacitive coupling electric field (CCEF) treatment (8 h/day for 90 days, 12.5 Hz signal, sinusoidal wave 60 kHz, attached by conductive adhesive pads paraspinally on the skin) in vertebral compression fractures (Piazzolla et al., 2015).

#### Neurological wounds

ES also has a number of neurotherapeutic applications. *1. CNS damage*. In early stages of Central Nervous System (CNS) damage, natural neuronal repair is often initiated, but fails to overcome tissue distortion and progressive pathologies, leading to neuronal degeneration and death (Harel and Strittmatter, 2006; Vajn et al., 2013). However, spinal cord neuronal regeneration in human clinical trials of 14 patients (Shapiro et al., 2005; Shapiro, 2012) have indicated promising results using implanted oscillating field stimulation. This reverses the polarity of the applied field being exposed to the injured axons every 15 min to deliver a field of 500–600 μV/mm, promoting a bidirectional axonal regeneration (Hamid and Hayek, 2008), with a measure of restoration of both sensory and motoric function. Moreover, in spinal cord injury (SCI) the unused paralyzed muscles undergo atrophy, and the lack of exercise leads to cardiovascular problems. This can be ameliorated by functional electric stimulation (FES). In contrast to ES stimulation in non-excitable tissues, with FES transcutaneous or implanted electrical stimulation pulses trigger action potentials in the intact peripheral nerves to generate muscle contraction. This in turn improves muscle bulk, stimulating the peripheral circulation and cardiovascular conditioning (Hamid and Hayek, 2008). In a systematic review of FES treatment of tetraplegic SCI survivors, the five studies that met the inclusion criteria all reported some improvements in function (Patil et al., 2015). In a pilot study of two tetraplegic SCI patients, FES is also found to improve hand motor function (Lu et al., 2016). From a rodent model it is also suggested that ES may attenuate apoptosis (cell death) and provide other neuroprotective effects in SCI (Zhang et al., 2015). *2. Peripheral nerve damage*. In injured peripheral nerves, recovery is often disappointing, due to poor regenerative capacity of neurons. Promisingly however, electrical stimulation enhances nerve regeneration, targets re-innervation and improves functional recovery in animal and human models (Gordon, 2016). *3. Post-stroke rehabilitation*. In stroke treatment, neuromuscular electrical stimulation (NMES) is applied to peripheral nerves or motor points of target muscles in order to restore motor function. With NMES application recovery is improved compared with controls in numerous (although not all) studies involving upper and lower limb and hand rehabilitation (reviewed by Knutson et al., 2015, 2016).

#### Myocardial wounds

Myocardial infarction results in wounding to heart tissue, and is a leading cause of death globally (Bui et al., 2011). Strategies for regeneration of the heart include stimulating the remaining live cells in the heart, and cardiac tissue engineering to provide replacements for diseased or damaged tissues (Ptaszek et al., 2012; Thavandiran et al., 2013). Electrical stimulation has been demonstrated to promote a remarkable ultrastructural organization of cardiomyocytes in animal models (Radisic et al., 2004). However, the effects of electrical field stimulation in human cardiac tissue engineering remain unreported in the literature and recapitulation *in vivo* of the key signals that influence cells to develop appropriate structure and associated function remains elusive (Thavandiran et al., 2013).

#### The eye

A unique pattern of electric currents is found in the eye lens, with large outward currents (20–40 μA/cm^2^) at the lens equator and inward currents at the anterior and posterior poles (Robinson and Patterson, 1982). This may have a basis in the spatial distribution of Na^+^/K^+^-ATPase activities in the lens epithelial cells (LECs; Candia and Zamudio, 2002). Following cataract extraction, an artificial lens is often implanted, on the premise that the human lens has no capacity for regeneration. Unfortunately, after cataract surgery, aberrant proliferation sometimes results in posterior capsule opacification (PCO; Zhao et al., 2012).

However, when the anterior capsule is preserved, the lens does indeed regenerate. LECs *in vivo* migrate either toward the equator, or toward the front of the lens. Given that applied EFs direct the migration and differentiation of cultured LECs, this suggests that the endogenous lens EF may be directing the LEC migration and differentiation *in vivo*. Moreover, LECs differ in their response according to their location in relation to the EF vector. PCO occurs due to inappropriate proliferation and migration of LECs, possibly as a result of the normal electric control of migration and proliferation being disrupted following surgical lens removal. It has been proposed that preservation of the whole capsule with its attached anterior lens preserves the electrical signals which may be critical to lens regeneration (reviewed by Zhao et al., 2012).

A TEP also exists in the retinal pigment epithelium (Miller et al., 1978; Griff et al., 1985; Quinn and Miller, 1992). Degenerative diseases of the retina, such as macular degeneration, exhibit numerous channelopathies, in which there is a reduced number or malfunction of membrane ion channels (Wimmers et al., 2007; Zhao et al., 2012). The importance of the retinal TEP is suggested by inactivation of a gene that abolishes the TEP in mice, which leads to a phenotype similar to retinitis pigmentosa in humans (Bosl et al., 2001).

The wound-generated voltage changes are transduced into second-messenger cascades and changes of gene expression via, for instance, activation of voltage gated ion channels, and voltage regulation of phosphorylation, which lead to functional changes in proteins (Okamura and Dixon, 2011; reviewed by Tseng and Levin, 2013). The first genes involved in this signal transduction have been identified by Zhao et al. (2006). The directional migratory response of cells depends on the opposing, “compass sensing” activity of two enzymes: *PIP3* kinase (Phosphoinositide 3-kinase) is polarized to the leading edge and the lipid phosphatase *PTEN* (phosphatase and tensin homolog) to the trailing edge (Stewart et al., 2007). Genetic disruption of *PIP3* impairs the KC electrotactic migration, and deletion of the *PTEN* gene enhances it (Zhao et al., 2006).

#### ES and other therapies in combination

Since both bioelectric and pharmacological interventions have shown a measure of success in clinical wound healing applications, promising future therapies could employ combined electric-pharmacological strategies (Martin-Granados and McCaig, 2014). For instance, discovery of the genes (encoding *PI3K* and *PTEN*) that regulate electrically driven wound healing have led to the possibility of *PTEN* inhibition as a pharmacological target to enhance wound healing (Zhao, 2007). Other combined interventions are also promising. For instance, ES can contribute to improved healing of pressure ulcers compared with standard wound care, but may be most effective in combination with other techniques such as vacuum-assisted closure (VAC) therapy and the application of platelet-rich plasma (PRP; Kuffler, 2015).

A number of pilot studies feature promising ultrasound applications. For instance, non-healing diabetic foot ulcers treated with ultrasound three times per week exhibited significant wound area reduction (Yao et al., 2014), by reducing inflammatory cytokines and facilitating tissue regeneration. In venous stasis ulcerations, ultrasound was found to improve wound healing by stimulating cell proliferation (Samuels et al., 2013). Improved wound healing using ultrasound is reported for critical limb ischemia (Kavros et al., 2007). An ultrasound variant involving surface acoustic wave (SAW) patch therapy led to improved tissue oxygenation in ischaemic feet (Rosenblum et al., 2014). Treatments involving combined ultrasound and electric field stimulation (CUSEFS) appear to complement and supplement one other (Avrahami et al., 2015), leading to improved closure of intransigent diabetic foot ulcers and venous leg ulcers. The ultrasound stimulates fibroblasts to create collagen, and collagen deposition becomes more organized, providing an improved weave for epithelialization. The electric stimulation pulls the fibroblasts and collagen together, resulting in a tighter and more ordered collagen weave overall. However, further high quality trials are required, as indicated by the mixed success (based on only low quality evidence to date) of electrotherapy and ultrasound rotator cuff disease treatments (Page et al., 2016). Moreover, the success rate of treatments such as PEMF appears to vary dramatically due to differing parametric settings and treatment strategies (Shi et al., 2013).

## Outlook

In relation to wound healing technologies, application of an electric field in clinical situations increases the rate and success of wound repair, with the largest evidence base in favor of unidirectional ES (Koel and Houghton, 2014). However, the underlying mechanisms are still not well-understood (Koppes et al., 2014). Regarding regeneration, although electrical field stimulation invokes regeneration in animal models, its application for human regenerative tissue technologies remains in its infancy, hindered by the elusive nature of the key regenerative mechanisms and pathways which could be translated into clinical outcomes (Eming et al., 2014). Moreover, knowledge that has been acquired of genetic and protein data implicated in regeneration remains to be coupled to how regenerative growth emerges from cellular activities (Lobo et al., 2012). In relation to this, three findings emerge as promising but little studied areas of research to shed light on these problems, as follows.

### Mapping of voltage patterns in body patterning and wound healing

Bioelectric parameters (such as *V*_*mem*_ and electric fields; Section Bioelectric Mechanisms of Wound Healing and Regenerative Processes in Animals and Plants—An Overview) predict and correlate both with growth/patterning and wound healing/regeneration. Yet large gaps remain. For instance, what is the relationship *between the two*, viz. the electric fields associated with external morphology and those associated with wounding and regeneration? For instance, voltage patterns conform to complex tree morphology (Rosene and Lund, 1953); and yet the bioelectric parameters of tree wound calli (which appear to regulate their development in relation to the natural form of the tree) remain unknown.

A hypothesis emerging from this data is that these bioelectric features bear instructive information (Levin, 2013) which are involved in *ultimately conforming the wound tissues to the morphology of the organism*. This is consistent with the hypothesis that electric currents play a role in *Xenopus* tadpole tail regeneration (Reid et al., 2009). Similarly, in earthworm regeneration, each segment has a specific electric potential. Segments are added by regeneration until the total endogenous field potential is that of a normal full-sized worm (Kurtz and Schrank, 1955; Levin, 2009), suggesting that bioelectric cues are essential to the regeneration process, which fundamentally recapitulates the generation of body form. Secondly, if electric fields are indeed implicated in regeneration processes, voltages imposed within the physiological range can be expected to stimulate regeneration. This is indeed the case. Applied fields drive regeneration of limb morphology in amphibians, birds and mammals (Section The Role of Ionic Flux Changes in Highly Regenerating Animals). With electric field stimulation, crop plant cell cultures exhibit improved growth rate and shoot formation (Goldsworthy and Rathore, 1985), including plants recalcitrant toward regeneration (Gill et al., 1987). Applied MFs stimulate kiwi shoot regeneration from callus (Rugini et al., 1991). Pulsed magnetic field (PMF) applied to soybean seedlings stimulate a higher frequency of shoot and root regeneration (Radhakrishnan and Kumari, 2013). Magnetic fields (MFs) applied to the commercially important *Paulownia* explants display increased regeneration percentages, shoot numbers, and chlorophyll content, with the authors speculating that MFs directly interact with Ca^2+^- channel proteins and the movement of Ca^2+^- into the cytosol, which in turn may lead to the production of cytokinin, an adenine-derived plant hormone which stimulates protein synthesis and cell division (Çelik et al., 2008). From these evidences it appears that bioelectric signals are indeed providing instructive information in the initiation, proliferation, differentiation and integration of new tissues, and the orchestration of the morphogenetic form appropriate for the wound region and characteristic to the organism.

The discovery of ion channels and pumps such as V-ATPases, found ubiquitously in animals and plants, and their role in activating downstream morphogenetic cascades is particularly salient. Since bioelectric determinants of morphology are largely invisible to modern molecular profiling techniques, Levin and colleagues advocate mapping between spatiotemporal ionic profile patterns and tissue patterning outcomes (Levin, 2012b). This same general rationale can be applied not only for morphogenesis, but also for regeneration. This is a strategy aimed at discovering the precise identification of the bioelectric information that activates the regeneration processes.

**Figure 3:**
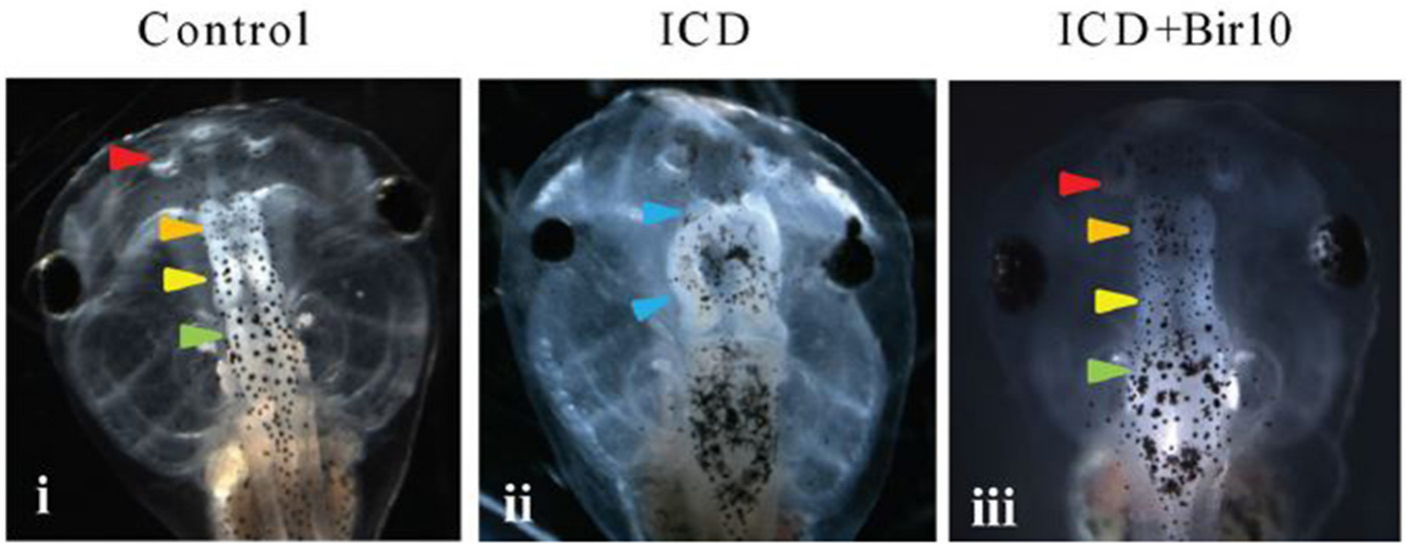
Effect of *V*_mem_ disruption on brain development in *Xenopus* embryos, and subsequent rescue by forced hyperpolarization. GFP-labeled to show subsequent developing brain structures at Stage 45. **(i)** Control, showing normal development of nostrils, olfactory bulb/forebrain, midbrain, and hindbrain (arrows). **(ii)** Tadpoles injected with constitutively active *notch ICD at 4-cell stage* showed severely malformed neural patterning with absent forebrain and a mispatterned midbrain at Stage 45 (blue arrowheads). **(iii)** Tadpole injected with both *Notch ICD* and hyperpolarizing *Bir10* ion channel (leading to forced hyperpolarization) showed restoration of neural patterning with intact nostrils and distinct forebrain, midbrain, and hindbrain [arrowhead colors as in panel **(i)**]. (From Pai et al., 2015, reprinted with permission).

However, the generative processes underlying morphogenesis are still not well-characterized, and remain elusive, with numerous reviews focusing on this problem (e.g., Wardlaw, 1970; Raff and Kaufmann, 1983; Goodwin, 1985, 2000; Gordon and Parkinson, 2005; Levin, 2012a; Newman and Linde-Medina, 2013; Tyler, 2014). Voltage mapping provides some of the most tractable and exciting methodologies to test the above hypothesis, i.e., if there is indeed a relationship between the bioelectric features of wounds and the morphology of the organism, and potentially provides a handle on any mechanisms in common. Moreover, a recent application of voltage mapping indicates its promise for clinical interventions. On the basis that transmembrane potential (*V*_*mem*_) disruptions lead to brain malformations in amphibian models, which can be rescued by voltage modulations (Pai et al., 2015; Figure 3), such knowledge could be translated into procedures to apply ion channel drugs. Such drugs, already approved for human use, could promote the required changes in *V*_*mem*_ properties to improve the wound healing response. There is growing evidence to show the relevance for such voltage modulation as a therapeutic target, based on comparison of animal and human model systems. For instance, a microarray genome-wide analysis combined with pharmacologically induced ion channel depolarization leads to numerous transcriptional responses, which are conserved between diverse model systems (frog, axolotl, and human), in *in vivo* and *in vitro* contexts, and affecting various disease networks in common, suggesting a conserved set of responses (Pai et al., 2016).

However, currently only ES applications are in clinical use. The clinical application of voltage modulations remain in their infancy, applied to elucidating bioelectric pathways only in the animal model system. Such studies hold great promise for informing a much more effective control of regenerative growth in biomedical settings (Adams and Levin, 2013).

### Current reversals

What causes the current switch from positive to a negative polarity in highly regenerating animals? Although, the molecular basis for this reversal is unknown, such reversals can be found elsewhere in the animal and plant kingdoms, allowing its basis to be explored in more accessible model systems, such as the plant root tip. Thus, another testable hypothesis is that this phenomenon in regenerating animals may have mechanisms in common with the plant root apex transition zone. The electric current peaks and oscillates in this zone (Masi et al., 2009) and is also synchronous with oscillating gene expression patterns (Moreno-Risueno and Benfey, 2011). It would be interesting, therefore, to further probe the relationship between the current reversals and various gene-expression patterns and their place in the instructional pathways guiding growth in this region.

Given the link between underlying bioelectric processes common to morphogenesis, wound healing and regeneration demonstrated above, another relevant model system to probe the basis for ion flux changes is the limb bud, in which there is a current switch from inward to outward three stages before limb bud formation, which predicts the site of the limb-forming region (Altizer et al., 2001).

### Aberrant and quiescent voltage patterns

**Figure 4:**
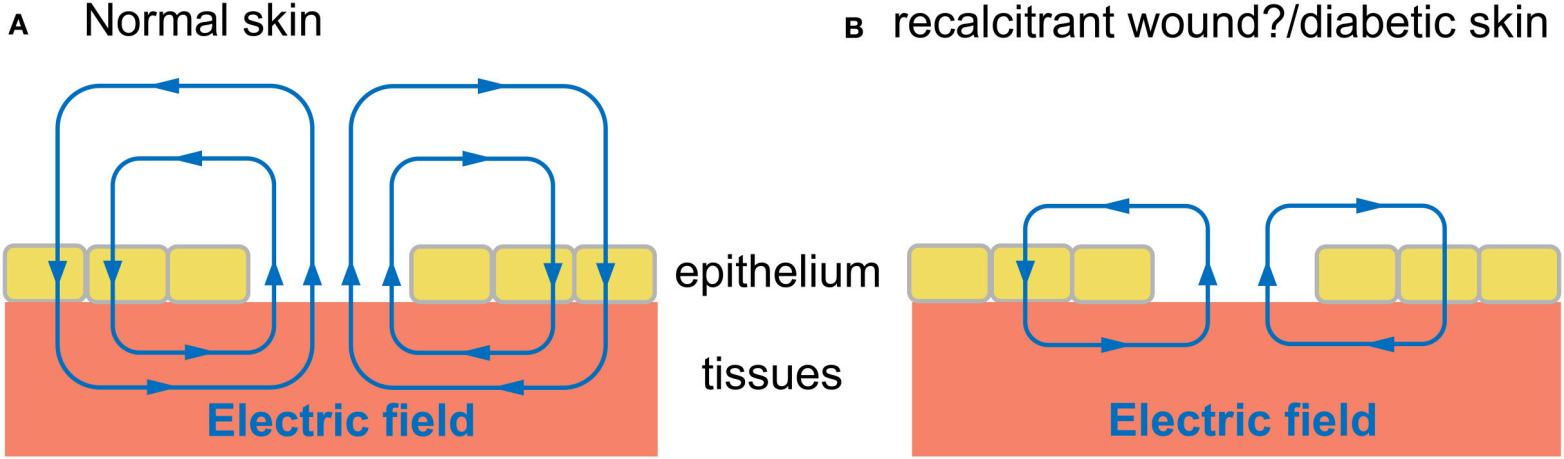
Proposed model of electric field during wound healing in normal skin compared with diabetic skin and wounds recalcitrant to healing. Hypothetical schematic diagram (adapted from McCaig et al., 2005). Electric field (blue lines) in tissues and wound bed, with arrows indicating direction of field lines. The “wound current” flows in the same direction of arrows, to drive migration of cells into wound bed, promoting wound healing. **(A)** Electric field of normal skin wound. **(B)** Wound of diabetic skin, aged normal skin, and other recalcitrant wounds may have a diminished electric field and associated wound current, which could explain the impaired wound healing in these various patients.

In recalcitrant wounds, it has been suggested that the endogenous EFs are askew, compromised or absent (Kloth, 2014; Fraccalvieri et al., 2015). Moreover, diabetic skin has a lower TEP and therefore smaller wound currents (Ionescu-Tírgovişte et al., 1985). The TEP in normal skin also declines with age (Nuccitelli et al., 2011), which could be a contributory factor in age-related delayed wound healing and impaired microcirculation. These ideas are summarized in a model (Figure 4). The figure shows the field lines have a directionality indicated by arrows, and the current also flows in the direction of the field lines. Compared with normal skin wounds (Figure 4A) the diabetic skin, aged normal skin and recalcitrant wounds (Figure 4B) exhibit a diminished electric field and associated wound current, which could explain the impaired wound healing in these various patients. Further research is required to experimentally verify this model which is still largely unexplored. This is in spite of promising findings, such as in aged skin, in which electrotherapy (Section Clinical Applications) can promote wound closure and reverse the ischaemia (reviewed by Gould et al., 2015). This is potentially of great importance if the endogenous currents are indeed aberrant, because this could be a focus for more informed therapeutic interventions. For instance, Cl^−^ and Ca^2+^ fluxes are dynamically regulated during the wound response in normal epithelium (Vieira et al., 2011) but have not yet been elucidated in non-healing ulcers. Therapeutic interventions could then target and normalize impaired specific ion fluxes that underlie effects such as diminished cell migration into the wound bed. This rationale is further confirmed by empirical findings from a diabetic mouse model, in which the cornea expresses weaker wound currents. This correlates with impaired wound healing and, importantly, is attributed at least in part to diminished flux of Cl^−^ ions and lower expression of anion transporters (Shen et al., 2016).

This kind of knowledge could also inform the design of next generation electroceutical wound care devices. These could incorporate graphene which, with its extreme electrical conductivity and sensititivity to magnetic fields (Salcman, 2014), may have numerous applications for non-contact electric stimulation (Mattei and Rehman, 2014), by providing sufficient field strength to control cell interactions (Heo et al., 2011).

More generally, in wound healing endogenous currents are known to activate several major signaling cascades, promote the directional migration of many cell types, and are involved in all three stages of wound healing (namely inflammation, new tissue formation, and remodeling; Martin-Granados and McCaig, 2014). Thus, it still remains to be explored precisely which of these processes are impaired in pathologies exhibiting aberrant voltage patterns, and their causal relationship in either generating or reflecting the pathologies. Moreover, although the human ability to regenerate finger tips correlates with the existence of an endogenous current emerging from the stump, the role of this current still remains to be discovered. The ability to regenerate digit skeletal structures in humans is restricted to the terminal phalangeal bone (Neufeld and Zhao, 1995) and, at present, there remain insurmountable technical problems for regrowing human digits and limbs in the laboratory (Shieh and Cheng, 2015). The closest experimental counterpart is the rodent model. However, although the anatomical and molecular events of digit regeneration in mice are becoming increasingly understood (Han et al., 2008), the role of bioelectric cues in these processes still largely awaits to be investigated, even in this mammal model. Such studies could help to close the gap between the clinical need and the basic science.

Plants and plant extracts appear to have therapeutic potential, with 21,000 plants so listed by the World Health Organization (WHO). Many of these are targeted to wound healing or neuroprotection (Ghosh and Rangan, 2012; Sharma et al., 2013. For instance, *Alpinia oxyphylla* fruits afford neuroprotective effects against glutamate-induced wounding to cortical neurons Yu et al., 2003). Most studies to date are applied on animal models such as mice and rats, and further human studies are required to determine their target modes of action and effectiveness. A third testable hypothesis is proposed, that such phytopharmaceuticals act by augmenting or restoring aberrant wound currents in recalcitrant wounds.

### Patterns in organisms of elevated longevity

Certain organisms have extremely long lifespans, such as multi-centennial rougheye rockfish, *Sebastes aleutianus* (lifespan ~200 years; Cailliet et al., 2001); the Greenland shark (*Somniosus microcephalus*; at least 272 years; Nielsen et al., 2016); and certain bivalve molluscs such the clam *Arctica islandica*, with a lifespan of up to 507 years (Butler et al., 2013). In such organisms virtually all of their bodily functions ranging from brain to heart and immune system are exquisitely preserved (Austad, 2010), and thus their study may help to unravel the primary causes and molecular patterns of age-related cellular degeneration (Philipp and Abele, 2009). Moreover, wound healing and regenerative capacities are known to be extraordinarily elevated in such animals (Philipp and Abele, 2009), so it would be interesting to ascertain whether this can be attributed to any bioelectric characteristics (such as *V*_*mem*_). These are as yet undocumented. Such data could also be contrasted with that of aberrant wound patterns (Section Aberrant and Quiescent Voltage Patterns) to seek clues as to mechanisms which in aberrant patterns are impaired, and in exceptionally long lifespans may be augmented.

Longevity in plants can exceed even that of extremely long-lived animals, with *Pinus longaeva* known to live from 4,713 to 5,062 years (Lanner and Connor, 2001; http://www.rmtrr.org/oldlist.htm) However, compromised regeneration in aged trees is a serious problem in horticulture, limiting the propagation of elite cultivars (Ikeuchi et al., 2016). Reduced root regenerative capacity has been attributed to loss of auxin responsiveness in peas (*Pisum sativa;* Rasmussen et al., 2015), whilst application of auxin improves regeneration ability in aged plants of *Arabidopsis* (Chen et al., 2014). Moreover, regeneration frequency is increased with an imposed electric field, and this correlates with an increase of auxin concentration (Kral et al., 2016). Wound-induced gene expression is tightly correlated with wound-induced electrical signal transmission in *Arabidopsis* (Mousavi et al., 2013; Forde and Roberts, 2014), and these signals lead to the upregulation of the jasmonate pathway (Mousavi et al., 2013; Section Bioelectric Mechanisms of Wound Healing and Regenerative Processes in Animals and Plants—An Overview). It is also recognized that jasmonate and auxin signaling pathways interact at various levels (Larrieu and Vernoux, 2016), whereby jasmonate can promote auxin biosynthesis, and auxin can induce the expression of jasmonate biosynthetic genes (Zhang et al., 2016). Auxin is both a versatile messenger in cell interactions, and provides patterning information (reviewed by Berleth and Sachs, 2001). Activation of auxin signal transduction genes leads to downstream cascades, which activate auxin responsive elements, which finally mediate the gene regulation involved in cell division and expansion (De Vos et al., 2012). Thus, taken together these findings suggest that it would be productive to probe the characteristics of wound-induced electric signals in aged plants, to establish if any bioelectric factors contribute to the diminished regeneration capacity. This is not only of potential horticultural importance but may be of translational relevance if there is a disruption of common upstream signals and affected physiological processes, such as aberrant membrane voltages.

## Conclusion

This analysis of literature has demonstrated that bioelectric characteristics are crucial elements in normal development, wound healing and regeneration and occur ubiquitously throughout the animal and plant kingdoms. The nature of the molecular pathways involved in wound healing is fundamentally different between the kingdoms. However, common elements include electric field changes following wounding, and the involvement of various membrane ion channels and pumps. A central feature during regeneration is the re-instigation of the natural electric field patterns previously evident during morphogenesis. Importantly, applied electric fields are one of the very few strategies that have met with proven success in improving the healing rate of recalcitrant wounds, and in coaxing regeneration in organisms which do not normally regenerate. A recent conference on the molecular and cellular basis of regeneration and tissue repair aimed to provide an integrative platform for scientists using a wide variety of models, including plants (Galliot et al., 2017). Evidence was provided, for instance, of an electric field applied to *Arabidopsis* cut roots significantly increasing the regeneration rate (Kral et al., 2016). This example shows that plant studies are a contemporary focus of interest for regeneration studies, along with numerous animal models (Galliot et al., 2017), which together provide a growing evidence base of a role for bioelectricity which is of prospective translational relevance.

It is becoming increasingly recognized that the programme of development resides not only in the information of gene sequences within the transcriptional code (a combinatorial code of transcription factors), but also requires the involvement of other combinatorial sequence codes. These include the histone code (which multiplies the informational capacity of the genes); cell surface code (residing in cell surface glycoproteins); cytoskeleton code; apoptosis code and ubiquitin code (reviewed by Barbieri, 2016). Moreover, the concept of a simple, linear flow of information of gene sequences is giving way to new model of development involving biological networks, with a multi-directional flow of information moving between hierarchical tiers (Franklin and Vondriska, 2011). It is thus all the more remarkable that regeneration of body form and complex structures such as muscle and cartilage can be induced in the amputated limbs of animals that do not regenerate them, simply by the application of an electric field or a H^+^ efflux. This suggests that the bioelectric application has a master regulatory effect (Levin, 2009) to reactivate the developmental coding networks involved in regeneration.

Thus, the mapping of voltage patterns in normal/impaired healing/regenerating limbs, and discerning the cell and molecular signatures associated with them may translate into discovering the key players and signals activating these processes. Since we still do not know how the right molecules are orchestrated to be in the right place at the right time in either development or the shaping of wound healing and regeneration, the aberrant voltage pattern and the patterns of elevated longevity provide model systems to probe these mysteries. This can bring vital knowledge for future regenerative therapies.

## Author contributions

The author confirms being the sole contributor of this work and approved it for publication.

### Conflict of interest statement

The author declares that the research was conducted in the absence of any commercial or financial relationships that could be construed as a potential conflict of interest.

## Abbreviations

CNS: Central nervous system
EF: electric field
GLR: glutamate-receptor-like
ES: electrical stimulation
FES: functional electrical stimulation
LECs: lens epithelial cells
MFs: Magnetic fields
NMES: neuromuscular electrical stimulation
*PIP3* kinase: Phosphoinositide 3-kinase
PEMF: Pulsed Electromagnetic Fields
*PTEN*: phosphatase and tensin homolog
TEP: transepithelial potential
*V*_mem_: transmembrane potential.
